# Synergistic inhibitory effects of clopidogrel and rivaroxaban on platelet function and platelet‐dependent thrombin generation in cats

**DOI:** 10.1111/jvim.16727

**Published:** 2023-05-19

**Authors:** Sara T. Lo, Ronald H. L. Li, Catherine J. Georges, Nghi Nguyen, Cheyenne K. Chen, Claire Stuhlmann, Maureen Sigmund Oldach, Victor Noel Rivas, Samantha Fousse, Samantha P. Harris, Joshua A. Stern

**Affiliations:** ^1^ University of California Davis School of Veterinary Medicine William R. Prichard Veterinary Medical Teaching Hospital Davis California USA; ^2^ Surgical and Radiological Sciences University of California, Davis Davis California USA; ^3^ Surgical and Radiological Sciences University of California Davis School of Veterinary Medicine Davis California USA; ^4^ Medicine and Epidemiology University of California, Davis Davis California USA; ^5^ Medicine and Epidemiology University of California Davis School of Veterinary Medicine Davis California USA; ^6^ University of California Davis School of Veterinary Medicine – VME, UC Davis 2108 Tupper Hall, One Shields Avenue Davis, California 95616‐5270 USA; ^7^ Cellular and Molecular Medicine, College of Medicine University of Arizona Tucson Arizona USA; ^8^ Department of Medicine & Epidemiology University of California, Davis, 2108 Tupper Hall, One Shields Avenue Davis, California 95616 USA

**Keywords:** cardiology, cardiovascular, clopidogrel resistance, factor Xa inhibitor, hypertrophic cardiomyopathy, saddle thrombus, thromboembolism

## Abstract

**Background:**

Dual antithrombotic treatment (DAT) with clopidogrel and rivaroxaban sometimes is prescribed to cats with hypertrophic cardiomyopathy at risk of thromboembolism. To date, no studies have evaluated their combined effects on platelet function.

**Objectives/Hypothesis:**

Evaluate the safety of DAT in healthy cats and compare, ex vivo, platelet‐dependent thrombin generation and agonist‐induced platelet activation and aggregation in cats treated with clopidogrel, rivaroxaban, or DAT. We hypothesized that DAT would safely modulate agonist‐induced platelet activation and aggregation more effectively than single agent treatment.

**Animals:**

Nine apparently healthy 1‐year‐old cats selected from a research colony.

**Methods:**

Unblinded, nonrandomized ex vivo cross‐over study. All cats received 7 days of rivaroxaban (0.6 ± 0.1 mg/kg PO), clopidogrel (4.7 ± 0.8 mg/kg PO), or DAT with defined washout periods between treatments. Before and after each treatment, adenosine diphosphate (ADP)‐ and thrombin‐induced platelet P‐selectin expression was evaluated using flow cytometry to assess platelet activation. Platelet‐dependent thrombin generation was measured by fluorescence assay. Platelet aggregation was assessed using whole blood impedance platelet aggregometry.

**Results:**

No cats exhibited adverse effects. Of the 3 treatments, only DAT significantly decreased the number of activated platelets (*P* = .002), modulated platelet activation in response to thrombin (*P* = .01), dampened thrombin generation potential (*P* = .01), and delayed maximum reaction velocity (*P* = .004) in thrombin generation. Like clopidogrel, DAT inhibited ADP‐mediated platelet aggregation. However, rivaroxaban alone resulted in increased aggregation and activation in response to ADP.

**Conclusion and Clinical Importance:**

Treatment combining clopidogrel and rivaroxaban (DAT) safely decreases platelet activation, platelet response to agonists, and thrombin generation in feline platelets more effectively than monotherapy with either clopidogrel or rivaroxaban.

**Abbreviations**
ADP: adenosine diphosphate
CAM: derivatized clopidogrel active metabolite
CATE: cardiogenic arterial thromboembolism
EDTA: ethylenediaminetetraacetic acid
HCM: hypertrophic cardiomyopathy
HPLC: high‐performance liquid chromatography
MFI: median fluorescence intensity
MYBPC: myosin binding protein C gene
PPP: platelet‐poor plasma
PRP: platelet‐rich plasma

## 1 INTRODUCTION

Hypertrophic cardiomyopathy (HCM), characterized by concentric left ventricular hypertrophy and diastolic dysfunction, is the most common cardiac disease in cats and affects approximately 15% of the general feline population.^1^, ^2^ Cardiogenic arterial thromboembolism (CATE), a devastating complication that occurs in 6%‐17% of cats with underlying cardiomyopathies, often carries a poor prognosis with a high mortality rate of up to 67%.^3^ It occurs when an intracardiac thrombus, most commonly formed in the left auricle, embolizes to an artery and impedes blood flow, leading to tissue ischemia and ischemic reperfusion injury.^4^ In 90% of cases, CATE results in acute distal aortic occlusion, also known as a saddle thrombus.^4^ For that reason, CATE is a very distressing emergency for owners and veterinarians because of the sudden occurrence of extreme pain, hindlimb or forelimb paralysis and often congestive heart failure without any forewarning. Despite a high mortality rate, thromboprophylaxis to prevent intracardiac thrombosis and recurrence of CATE is limited, and further research in this area is crucial.^4^, ^5^

Cats with HCM are hypercoagulable because of a pathologically enlarged left atrium promoting blood stasis and endothelial injury.^6^, ^7^ This situation places cats with HCM at increased risk of CATE, with up to 11.3% of cats developing CATE within 10 years of HCM diagnosis.^8^, ^9^ Other outcomes of HCM in cats include a persistent subclinical state, left‐sided congestive heart failure (CHF), and sudden cardiac death.^8^ Although HCM affects domestic cats of any breed, sex, and age >3 months, purebred Maine Coon and Ragdoll cats homozygous for breed‐specific myosin binding protein‐C gene (*MYBPC3*) mutations develop severe HCM and are at higher risk for CATE.^10^

Although there is no universal standard of care exists for CATE, current guidelines recommend the use of clopidogrel, an antiplatelet drug that irreversibly inhibits the platelet adenosine diphosphate (ADP) receptor, P2Y_12_, to prevent CATE.^11^, ^12^ Previous studies showed that clopidogrel is well‐tolerated in cats and is superior to aspirin at decreasing recurrence rate and prolonging the time to recurrence of CATE.^13^ However, on‐treatment recurrence rate remained high at 36%, indicating that clopidogrel alone is not effective at preventing CATE.^13^ A plausible explanation for this finding is resistance to clopidogrel, reported in up to 15.4% of cats with HCM because of genetic polymorphism in the ADP receptor gene *P2RY1*.^11^, ^14^ Because of the variable response to clopidogrel and high mortality rate of CATE, a multimodal approach in thromboprophylaxis currently is employed in cats at risk of thrombosis at our institution. Administration of anticoagulant treatment including unfractionated heparin and low molecular weight heparin has been described in cats, but the required frequent SC administration of up to q6h compromises owner and patient compliance.^15^ Rivaroxaban is a direct PO anticoagulant drug that prevents clot formation by inhibiting activated factor X (factor Xa).^16^ In healthy adult cats, once daily administration of rivaroxaban is safe, well‐tolerated and effective at prolonging clotting times.^17^ In humans, dual treatment consisting of rivaroxaban and clopidogrel decreases the incidence of thrombosis in patients with coronary artery disease and is superior to single‐agent treatment in preventing acute cardiovascular events.^18^, ^19^ However, their synergistic effects on platelet function are poorly understood. In an in vitro study in humans, ticagrelor, a P2Y_12_ inhibitor similar to clopidogrel, enhanced the inhibitory effect of rivaroxaban on platelet‐dependent thrombin generation.^20^ A retrospective study in cats found favorable outcomes in those receiving dual antithrombotic treatment (DAT). The study found no first‐time occurrence of CATE after commencement of DAT and clinically unimportant bleeding adverse events in 15% of cats.^20^ However, little is known about the synergistic inhibitory effects of clopidogrel and rivaroxaban on platelets in cats.

Because rivaroxaban may not only improve owner compliance but also enhance the antiplatelet effects of clopidogrel, we sought to evaluate the safety and efficacy of DAT in healthy cats. We hypothesized that DAT with clopidogrel and rivaroxaban would safely modulate agonist‐induced platelet activation and aggregation more effectively than single agent treatment. To test our hypothesis, we aimed to (a) examine the safety of combining standard dose PO rivaroxaban with clopidogrel in cats and (b) compare, ex vivo, platelet‐dependent thrombin generation and agonist‐induced platelet activation and aggregation in cats treated with clopidogrel alone, rivaroxaban alone or DAT (clopidogrel and rivaroxaban).

## 2 MATERIALS AND METHODS

### 2.1 Subject selection

The study protocol was approved by the Institutional Animal Care and Use Committee of the University of California, Davis (IACUC protocol number 20565). Nine healthy cats (6 intact females and 3 intact males, 1 year of age) were selected from a colony of Maine Coon/outbred mixed domestic cats bred and raised at the UC Davis Feline HCM Research Laboratory. Of the 9 cats, 6 heterozygous and 3 wild‐type cats for the A31P variant in the myosin binding protein C gene (*MYBPC3*) gene were included. Cats were housed in group housing kennels and cared for by dedicated animal care technicians. Environmental enrichment was provided in the form of toys, boxes, shelves, and social interaction with cats and humans. Physical examination, CBC, serum biochemistry panel, and echocardiography performed by a board‐certified cardiologist (JAS) were performed to ensure all cats were clinically healthy before enrollment. Cats that were noncompliant with handling and restraint, had any echocardiographic evidence of HCM, hepatic or kidney disease, anemia (hematocrit < 25%), or thrombocytopenia (<150 × 10^9^/L) were excluded. Cats were monitored twice daily for adverse drug reactions including gastrointestinal signs and bleeding diathesis.

### 2.2 Drug administration

**FIGURE 1:**
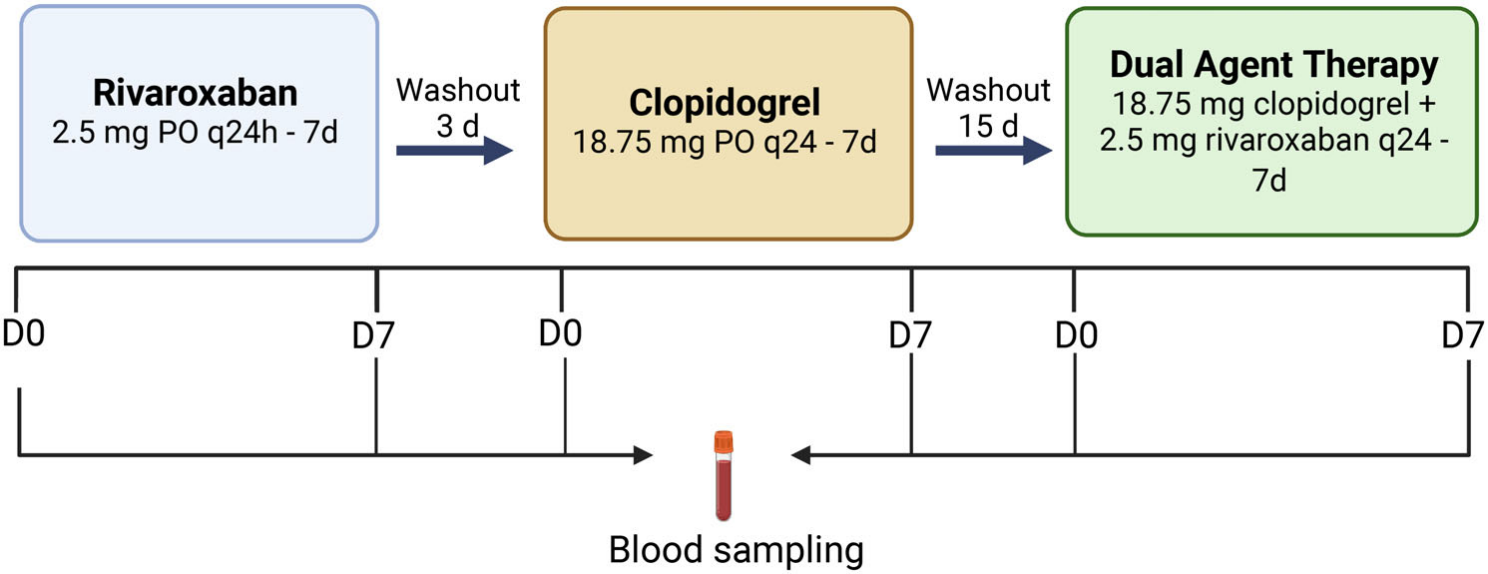
Flow diagram illustrating drug administration and blood sampling timeline. Clop, Clopidogrel; DAT, dual antithrombotic treatment; Riva, Rivaroxaban.

We utilized an unblinded, nonrandomized ex vivo cross‐over design. All cats received 7 days of rivaroxaban (2.5 mg PO q24h), clopidogrel (18.75 mg PO q24h), or DAT (Figure 1). The 3‐day and 15‐day washout periods after rivaroxaban and clopidogrel treatments, respectively, have proven to be sufficient for coagulation and platelet function to return to baseline.^17^, ^21^ Data measured during the study confirmed rivaroxaban assay and platelet aggregometry results had returned to baseline before advancing to the next treatment period. A sole researcher (STL) administered all medications to all cats to minimize variation. Citrated plasma samples, collected 3 hours after administration of rivaroxaban, were sent to the Cornell University Animal Health Diagnostic Center for measurements of plasma rivaroxaban concentration based on factor Xa inhibition. Cats did not receive any other medications for the duration of the study.

### 2.3 Blood sampling

Figure 1 outlines the blood sampling protocol utilized. All cats were sedated with 1‐2 mg/kg alfaxalone IM approximately 15 minutes before blood was obtained from the medial saphenous vein using a 21‐ or 23‐gauge butterfly needle. Samples then were immediately transferred to 3.2% trisodium citrate tubes (BD Vacutainer, Franklin Lakes, NJ) and micro heparin tubes (BD Microtainer, Franklin Lakes, NJ). Blood tubes then were inverted gently and inspected for clots. A maximum of 8 mL of blood per cat was collected on each sampling day. Blood samples on day 7 of the rivaroxaban and DAT trials were collected 3 hours after rivaroxaban administration to capture peak anti‐Xa activity.^17^ Blood samples on day 7 of clopidogrel treatment were collected 1.5‐2 hours after clopidogrel administration to capture maximum clopidogrel active metabolite (CAM) plasma concentration.^22^ Complete blood counts performed using citrated blood and an automatic analyzer (HM5 Hematology Analyzer, Abaxis, Abbott Group, Union City, CA). Heparinized whole blood was utilized once after the first blood collection for a biochemistry panel (VetScan V2 Chemistry Analyzer, Abaxis, Abbott Group, Union City, CA).

### 2.4 Whole blood impedance platelet aggregometry

Platelet aggregation was assessed using an automated whole blood impedance platelet aggregometer (Multiplate, Roche, Mannheim, Germany) according to manufacturer's instructions.^23^, ^24^ In brief, after 30 minutes of resting at room temperature, 300 μL of heparinized whole blood was added to pre‐heated test cells containing pre‐warmed 0.9% sodium chloride solution. After 3 minutes of incubation at 37°C under physiologic shear rate created by an 800‐rpm‐spinning Teflon‐coated magnetic stir bar, ADP (final concentration, 6.25 μM; MilliporeSigma, Burlington, MA) was added and electrical impedance was recorded for 6 minutes. Platelet aggregation, measured as electrical impedance over time on a pair of silver‐coated electrodes, was reported as velocity (AU/min), area under the curve (AUC; AU × min), and maximum aggregation (Ag max). Response to treatment was calculated based on the formula:

$$\%\;\text{Inhibition}=\frac{\left(\text{Aggregation}-\mathrm{pre}\right)-\left(\text{Aggregation}-\text{post}\right)\;}{\left(\text{Aggregation}-\mathrm{pre}\right)}\times 100\%$$

### 2.5 Measurement of platelet activation by flow cytometry

Flow cytometry was used to measure the degree of platelet activation in response to ADP or thrombin before and after each treatment. Citrated whole blood first was transferred to polypropylene tubes and incubated at 37°C for 30 minutes. Platelet‐rich plasma (PRP), generated by centrifugation (200 × *g*, 5 minutes, 21°C, no brakes), was analyzed within 2 hours of blood collection. Platelet rich plasma (PRP) then was standardized to 1 × 10^7^ cells/mL in 100 μL volume with Tyrodes‐HEPES buffer (5 mM dextrose, pH 7.2, without divalent cations). Platelets were either unstimulated (resting) or activated with 20 μM ADP (MilliporeSigma, Burlington, MA) or 0.005 units/mL bovine alpha‐thrombin (Haemtech, Essex Junction, VT; 37°C, 15 minutes) before addition of monoclonal rat anti‐mouse antibodies to P‐selectin conjugated with fluorescein isothiocyanate (1:200, clone RB40.34; BD Pharmingen, San Jose, CA) at 37°C for 45 minutes. Samples then were fixed with 1% paraformaldehyde. Flow cytometry was performed using a 5‐color flow cytometer (Beckman‐Coulter FC500, Miami, FL). Labeled platelets were identified by forward and side scatter properties, 0.9 μm and 3 μm calibration beads, and integrin beta‐3 (CD61) by allophycocyanin‐conjugated polyclonal mouse anti‐human antibody (1:1000, clone VI‐PL2; Invitrogen, Carlsbad, CA). Compensation was measured using monoclonal mouse immunoglobulin G1 kappa and anti‐mouse compensation beads conjugated to the matched experimental fluorochromes. Compensation matrices then were calculated using commercially available software (Flowjo, Treestar). To determine CD62P (P‐selectin)‐positive events within the platelet gate, gating boundaries were identified by fluorescence‐minus‐1 and isotype controls as previously described. The magnitude of platelet activation in response to ADP or thrombin was calculated based on surface expression of P‐selectin, measured as change in percent positive events or fold change in median fluorescence intensity (MFI) between resting and activated samples using the formula^11^, ^25^:

$$\mathrm{MFI}\;\text{fold change}\;\left(\log_{10}\right)=\left(\log_{10}\;{\mathrm{MFI}}_{\text{Activated}}\right)-\left(\log_{10}\;{\mathrm{MFI}}_{\text{Resting}}\right)$$

### 2.6 Platelet‐dependent thrombin generation

Citrated PRP was further analyzed to determine thrombin generation over time using a commercially available fluorescence assay (Technothrombin TGA, Diapharma, Westchester, OH) according to the manufacturer's instructions. Before analysis, all reagents were allowed to reach room temperature. Platelet rich plasma first was standardized to 1.5 × 10^8^ cells/mL with autologous platelet poor plasma, generated as described above, within 2 hours after collection. A 96‐well microtiter plate suitable for fluorescence measurement first was warmed to 37°C before the addition of 40 μL of PRP. Platelet rich plasma was maintained at 37°C before the addition of 1 mM fluorogenic substrate, N‐carbobenzyloxy‐Gly‐Gly‐Arg 7‐amido‐4‐methylcoumarin, in 15 mM calcium chloride and phospholipid micelles containing recombinant human tissue factor in Tris‐HEPES‐sodium chloride buffer. The plate was immediately analyzed at approximately 360 nm/460 nm (excitation/emission) after addition of the reagent and substrate mixture for 60 minutes in 1‐minute intervals. Each sample was analyzed in duplicate. Lyophilized human plasma, provided by the manufacturer, served as positive control. Thrombin generation was measured as time (seconds) to maximum thrombin generation (*T*_max_), peak thrombin generation (relative fluorescence unit; RFU), maximum velocity (*V*_max_, FI/s) and thrombin generation potential (AUC) over 1 hour.

### 2.7 Rivaroxaban assay

Plasma rivaroxaban concentrations were measured before and after 7 days of rivaroxaban and DAT. Platelet‐poor plasma was generated by centrifuging the remaining citrated blood after extracting PRP at 5000 × *g* for 10 minutes, aliquoted and stored at −80°C. Batched samples were analyzed using the Comparative Coagulation Laboratory at the Cornell University Animal Health Diagnostic Center for measurement of anti‐Xa activity chromogenic assay with a rivaroxaban calibration standard as described.^26^

### 2.8 Statistical analysis

Preliminary data based on a previous study was used to estimate the number of cats needed to achieve an approximately 33% decrease in platelet aggregation in response to clopidogrel treatment.^14^ With a 2‐tailed design, 9 cats were needed to detect significant changes with 80% power and a priori alpha of 0.05. Each cat served as its own control in order to determine the response to each of the 3 treatments. Normality was determined using the D'Agostino & Pearson normality test. Continuous variables were reported as mean ± SD or median and interquartile range (IQR), as appropriate. Pair‐wise comparisons of baseline and post‐treatment variables were performed using Wilcoxon matched‐pairs signed‐ranks tests or paired *t*‐tests, as appropriate. The 3 groups were compared using Kruskal‐Wallis or 1‐way analysis of variance (ANOVA) of repeated measures, as appropriate, followed by post‐hoc Dunn's or Tukey's multiple comparison tests, respectively. Categorical data among the 3 groups were compared using a Chi‐squared test. Correlations between plasma rivaroxaban concentrations, body weight and change in thrombin generation (% change = D0‐D7/D0 × 100) were evaluated using Spearman correlation. Statistical analyses were performed using commercially available software (Prism 8.0, GraphPad Software, San Diego, CA).

## 3 RESULTS

The mean dosages of clopidogrel and rivaroxaban administered were 4.7 ± 0.8 mg/kg and 0.6 ± 0.1 mg/kg, respectively. The mean weight of the 9 study cats was 4.1 ± 0.7 kg. None of the 9 cats exhibited adverse effects throughout the study period. All cats had rivaroxaban concentrations below the limit of detection (<25 ng/mL) before rivaroxaban or DAT. Only 2/9 cats (22%) achieved peak plasma concentrations >150 ng/mL after 7 days of rivaroxaban treatment. The median peak plasma rivaroxaban concentration was 108 ng/mL (interquartile range [IQR], 59‐258 ng/mL). Of the 9 cats, 1 (11%) cat achieved the proposed therapeutic range (>150 ng/mL) after 7 days of DAT. The median concentration after 7 days of DAT was 82 ng/mL (range, 17‐158 ng/mL). When rivaroxaban concentrations were corrected by body weight, high interindividual variability (coefficient of variation = 59.9%) was found among cats. Rivaroxaban concentrations also did not correlate with body weight (*r* = 0.3, 95% confidence interval [CI], −0.4 to 0.8; *P* = .4).

### 3.1 Adenosine diphosphate mediated platelet aggregation

**FIGURE 2:**
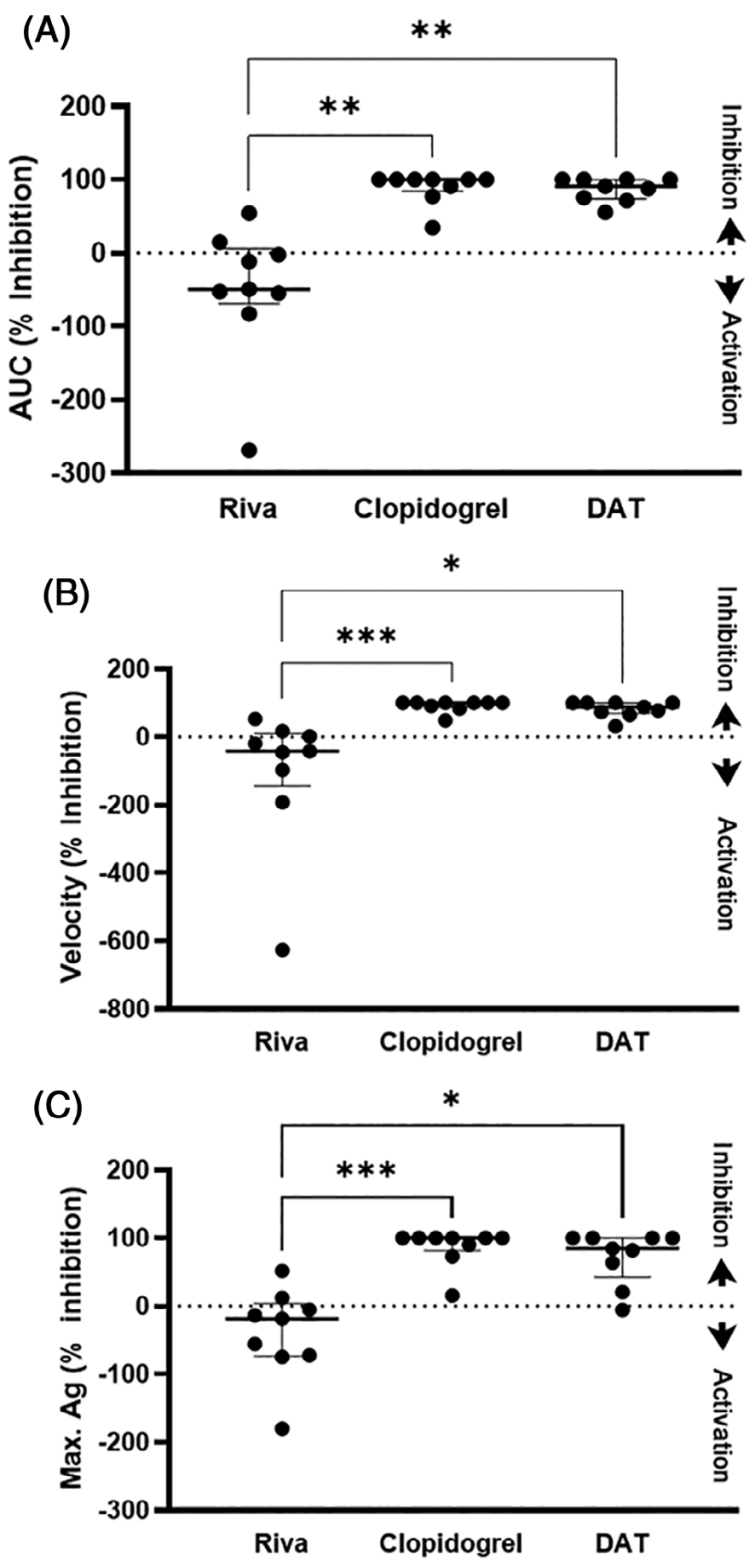
Scatter dot plots demonstrating platelet inhibition, measured as percent (%) inhibition, in 9 cats after 7 days of rivaroxaban (RIVA), clopidogrel, or dual antithrombotic treatment (DAT) with rivaroxaban and clopidogrel. ADP‐induced whole blood impedance aggregometry was measured as maximum aggregation (Ag max), velocity and area under the cure (AUC). Percent inhibition >0% indicates platelet inhibition (up arrows) and inhibition (%) < 0% indicates platelet activation (down arrows) after 7 days of treatment. Clopidogrel alone and DAT resulted in significant inhibition compared to Riva alone. Riva alone resulted in platelet activation in 7 of the 9 cats. Line represents median and error bars represent interquartile range. *P < .05; **P < .005; ***P < .001.

Figure 2 presents the effects of rivaroxaban, clopidogrel or DAT on ADP‐mediated platelet aggregation in whole blood. We found that clopidogrel alone (% inhibition, 100%; IQR, 84.3‐100) and DAT (% inhibition, 90.8%; IQR, 73.6‐100) had similar levels of inhibition in AUC (*P* > .99; Figure 2A). Both were significantly higher than rivaroxaban treatment alone (% inhibition, −18.4%; IQR, −73.6 to 3.6; *P* < .01). Similar patterns of inhibition were noted in aggregation velocity and maximum aggregation (Figure 2B,C). Of the 9 cats treated with rivaroxaban alone, 7 (77.8%) cats consistently had evidence of increased aggregation (inhibition levels <0%) in all 3 aggregometry results (AUC, velocity, and aggregation). These results were significantly higher when compared to clopidogrel (0/9, 0%) and DAT (1/9, 11.1%; *P* < .001; Figure 2). Table 1 summarizes the aggregometry results.

**TABLE 1:** Summary of ADP‐mediated whole blood platelet aggregometry in 9 cats receiving 7 days of rivaroxaban, clopidogrel or dual agent therapy.

	D0	D7	Inhibition (%)
Rivaroxaban	AUC = 80 (72.5‐144.5) Vel = 31.8 (16.2‐36.9) Ag_max_ = 150.4 (108.6‐233.4)	AUC = 118 (112.5‐130.5) Vel = 42.0 (33.9‐44.2) Ag_max_ = 186.4 (176.5‐208.0)	AUC = −49.4% (−68.8‐6.1) Vel = −42.1% (−144.8‐9.6) Ag_max_ = −18.4% (−73.6‐3.6)
Clopidogrel	AUC = 135 (107.5‐145.0) Vel = 38.2 (36.3‐42.4) Ag_max_ = 211.9 (168.7‐232.2)	AUC = 0 (0‐22.5)^a^ Vel = 0 (0‐5.1)^a^ Ag_max_ = 0 (0‐43.1)^a^	AUC = 100% (84.3‐100)^b^ Vel = 100% (87.3‐100)^b^ Ag_max_ = 100% (81.6‐100)^b^
DAT	AUC = 73 (50.5‐125.5) Vel = 23.5 (14.8‐30.9) Ag_max_ = 117.9 (74.0‐207.8)	AUC = 9 (0‐18.5)^a^ Vel = 2.9 (0‐7.4)^a^ Ag_max_ = 20.4 (0‐60.8)^a^	AUC = 90.84% (73.6‐100)^c^ Vel = 87.7% (69.3‐100)^c^ Ag_max_ = 85.0% (42.3‐100)^c^

*Note*: All data are presented as median (interquartile range).

Abbreviations: Agmax, maximum aggregation; AUC, area under the curve; DAT, dual agent therapy; Vel, velocity (arbitrary unit/min).

^a^ *P* < .05; D0 vs D7.

^b^ *P* < .05; Rivaroxaban vs Clopidogrel.

^c^ *P* < .05; Rivaroxabvan vs DAT.

### 3.2 Platelet activation by flow cytometry

**FIGURE 3:**
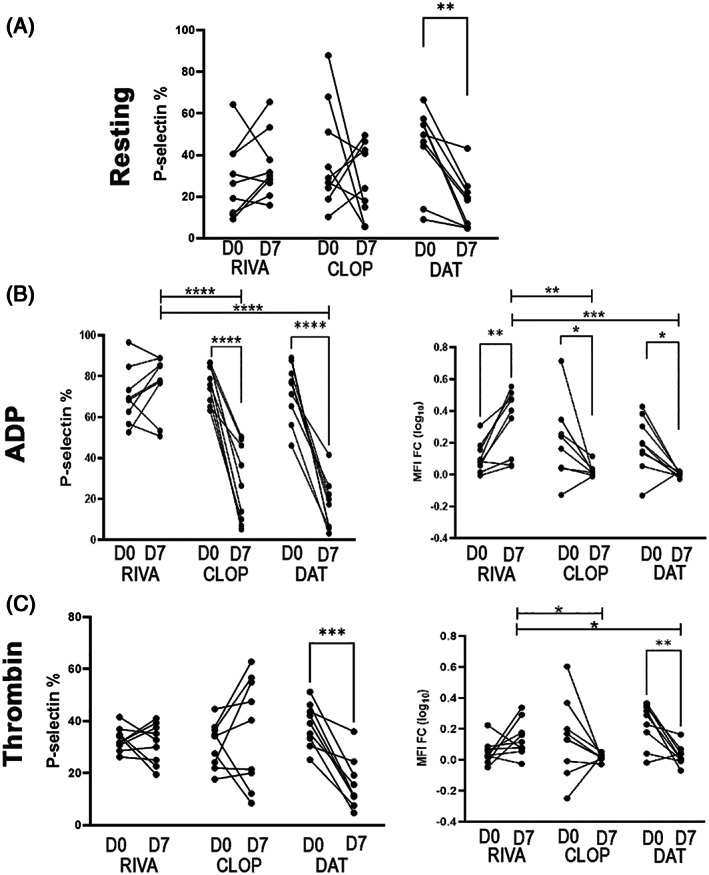
Scatter dot pots demonstrating platelet activation measured as number of P‐selectin positive platelets (%) or response to agonists, ADP or thrombin, measured as fold change in median fluorescence intensity (MFI FC log _10_), by flow cytometry in 9 cats treated with 7 days of rivaroxaban (RIVA), clopidogrel (CLOP) or dual antithrombotic treatment (DAT). (A) Only cats treated with DAT had significantly lower number of P‐selectin‐positive (%) resting (unstimulated) platelets compared to single agent treatment with RIVA or CLOP. (B) When platelets were treated with 20 μM ADP, CLOP or DAT significantly modulated response to ADP, while 7 days of RIVA treatment resulted in an increase response to ADP, which were significantly higher on D7 compared to CLOP or DAT. (C) Only DAT modulated platelet response to 0.005 U/mL thrombin, as shown by decreased percentage of P‐selectin‐positive platelets, and MFI FC from D0 to D7. Response to thrombin was significantly higher on D7 in cats treated with RIVA alone. *P < .05; **P < .005; ***P < .001; ****P < .0005.

Platelet activation in the presence or absence of ex vivo treatment of ADP or thrombin was characterized using platelet surface P‐selectin expression by flow cytometry (Figure 3). Baseline P‐selectin expressions measured on D0 were not significantly different (*P* = .3) before each treatment to ensure that all measurements returned to baseline before the next treatment. We found that in unstimulated platelets (resting), DAT resulted in a significantly lower level of P‐selectin‐positive platelets (D0 = 44.0% ± 19.6 vs D7 = 16.7% ± 12.8; *P* = .002; Figure 3A). When platelets were activated with ADP, rivaroxaban treatment alone led to an increased response to ADP (Figure 3B; MFI fold change log_10_ D0 = 0.11 ± 0.096 vs D7 = 0.33 ± 0.20; *P* = .003). This increased response to ADP after 7 days of rivaroxaban was significantly higher compared to the clopidogrel and DAT treated cats on D7 (*P* < .01; Figure 3B). As expected, clopidogrel not only led to lower numbers of P‐selectin positive platelets (D0 = 75.8% ± 9.0 vs D7 = 27.2% ± 18.9), but also modulated platelet response to ADP (D0 = 0.3 ± 0.3 vs D7 = 0.006 ± 0.02; *P* = .01). Similarly, DAT also inhibited response to ADP (D0 = 0.2 ± 0.2 vs D7 = 0.006 ± 0.03; *P* = .01; Figure 3B) as well as the number of P‐selectin positive platelets before and after DAT treatment (D0 = 44.02% ± 19.6 vs D7 = 16.7 ± 12.8%; *P* = .002). This suppression in response to ADP was similar between the 2 groups (*P* = .9).

When platelets were activated using thrombin, only DAT led to a decreased number of P‐selectin positive platelets (D0 = 38.6% ± 8.2 vs D7 = 15.7% ± 9.67; *P* = .0002), whereas rivaroxaban (D0 = 33.0% ± 4.56 vs D7 = 31.48% ± 7.66; *P* = .62) or clopidogrel (D0 = 31.07% ± 8.70 vs D7 = 36.09% ± 20.77; *P* = .44) alone did not modulate the number of activated platelets (Figure 3C). Similarly, platelet response to thrombin, calculated by MFI fold change (log_10_), was consistently decreased in cats before and after 7 days of DAT (D0 = 0.23 ± 0.14 vs D7 = 0.028 ± 0.064; *P* = .02). Whereas platelet response to thrombin did not change before and after 7 days of clopidogrel (D0 = 0.13 ± 0.25 vs D7 = 0.016 ± 0.022; *P* = .17) or rivaroxaban (D0 = 0.049 ± 0.077 vs D7 = 0.13 ± 0.12; *P* = .13), cats in the clopidogrel group on D7 showed similar response to thrombin when compared to DAT group (*P* = .9; Figure 3C).

### 3.3 Platelet‐dependent thrombin generation

**FIGURE 4:**
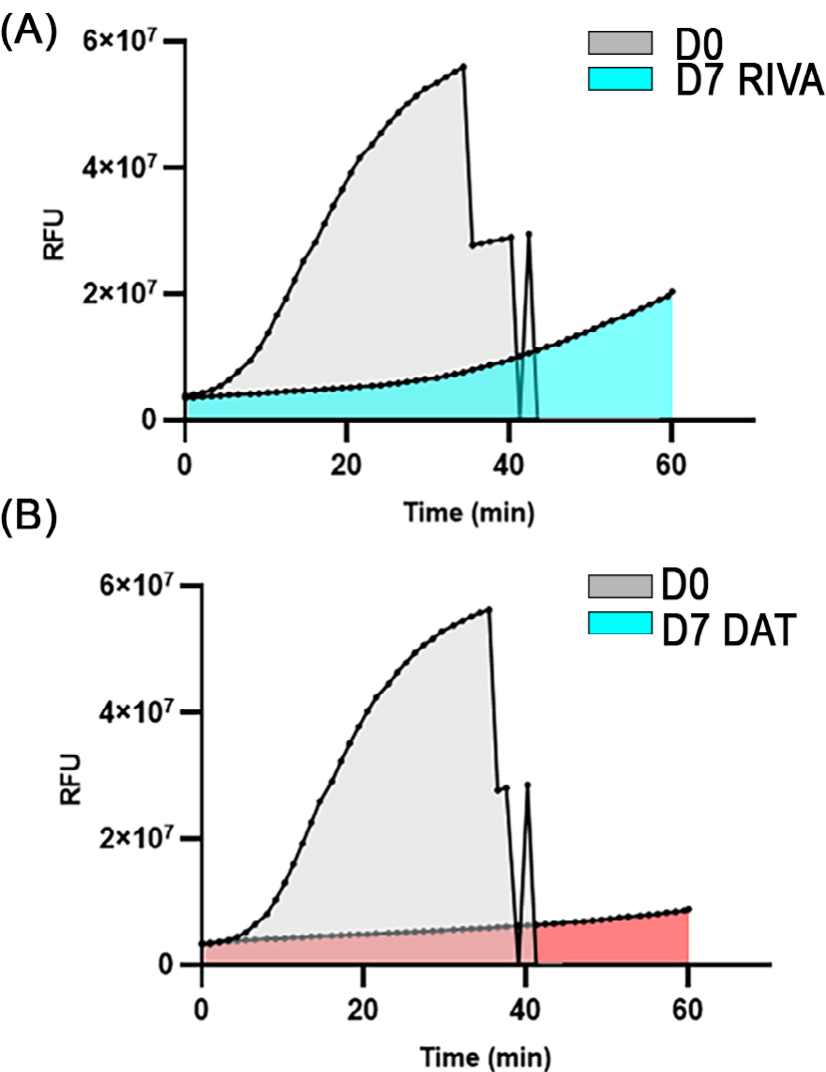
Representative thrombograms demonstrating continuous thrombin generation, measured as fluorescent signal in arbitrary unit (AU), in platelet rich plasma in a cat before and after 3 days of rivaroxaban treatment (RIVA) (A) and 7 days of dual antithrombotic treatment (DAT) with rivaroxaban and clopidogrel (B). Data points were derived from means of duplicate measurements at a single time point for 60 minutes. Note the flattening of thrombogram following DAT compared to rivaroxaban treatment alone.

**FIGURE 5:**
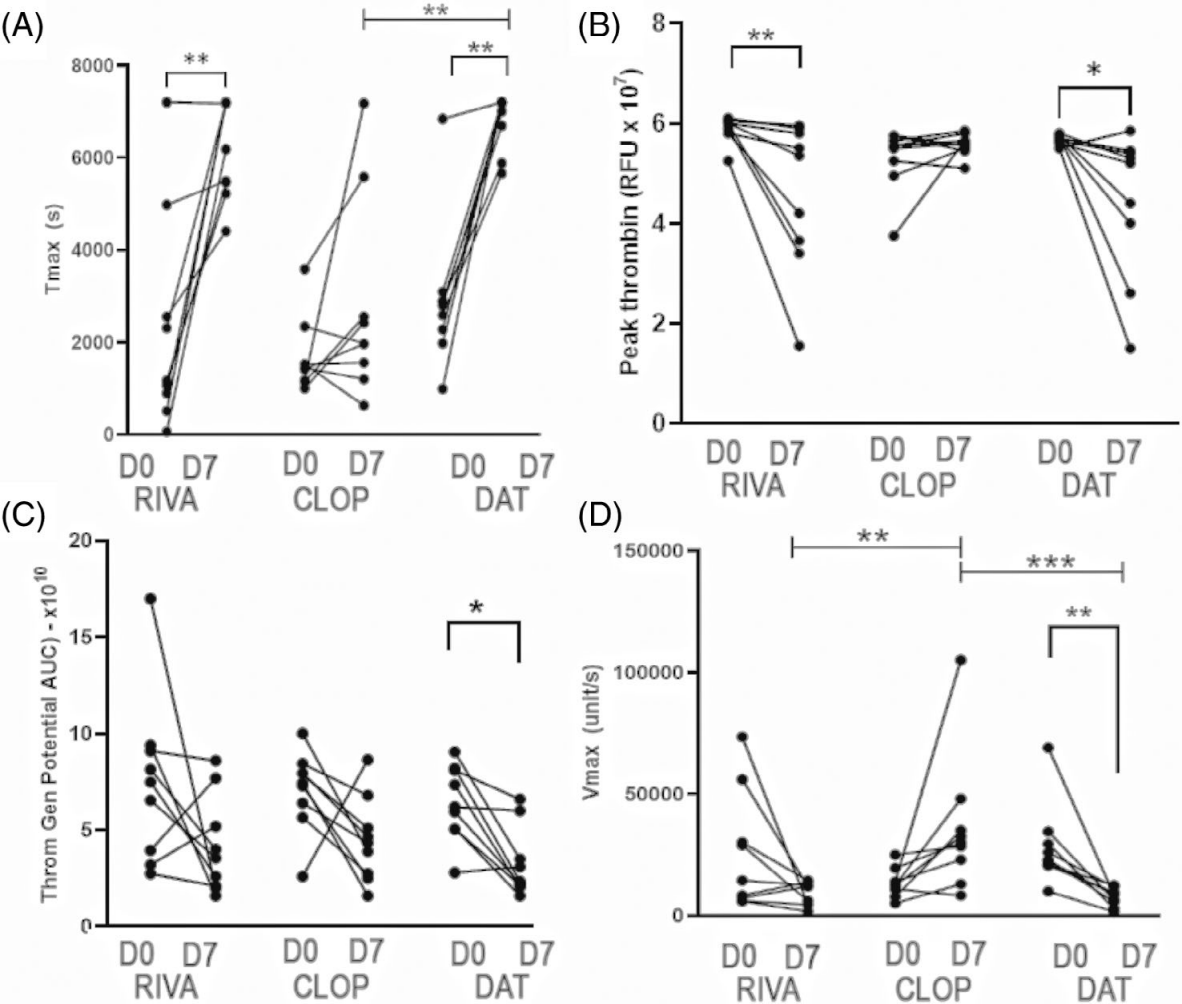
Scatter dot plots demonstrating platelet dependent thrombin generation over time in 9 cats, measured as (A) time (seconds) to maximum thrombin generation (*T*_max_), (B) peak thrombin generation (Relative Fluorescence Unit [RFU]), (C) thrombin generation potential (Area Under the Curve [AUC]) over 1 hour and (D) maximum velocity (*V*_max_, unit/s). After 7 days of treatment with rivaroxaban (RIVA), *T*_max_ and peak thrombin generation were significantly modulated from D0 to D7. In addition to *T*_max_ and peak thrombin generation, dual agent treatment (DAT) modulated thrombin generation potential and maximum velocity of thrombin generation from D0 to D7. Compared to RIVA, DAT also significantly prolonged *T*_max_ compared to clopidogrel (CLOP) alone on D7. *P < .05; **P < .005; ***P < .001.

Representative thrombograms of PRP in a cat receiving rivaroxaban and DAT are shown in Figure 4A,B, respectively. Rivaroxaban treatment significantly delayed time to maximum thrombin generation (*T*_max_) from 2306 ± 2352 s on D0 to 6165 ± 1065 s on D7 (*P* = .002; Figure 5A). Peak thrombin generation, measured as relative fluorescence intensity (RFU), was also significantly decreased (D0 = 5.88 × 10^7^; IQR, 5.8‐6.03 vs D7 = 5.35 × 10^7^; IQR, 3.53‐5.85, *P* = .004; Figure 5B). However, rivaroxaban treatment did not consistently modulate thrombin generation potential (AUC) (D0 = 7.511 × 10^10^ ± 4.35 vs D7 = 4.15 × 10^10^ ± 2.54, *P* = .11) or *V*_max_ (D0 = 25 632 ± 24 423 RFU/s vs D7 = 9088 ± 4792 RFU/s; *P* = .08; Figure 5C,D).

Similarly, DAT also prolonged *T*_max_ from 2795 s (IQR, 2129‐2993) on D0 to 7176 s (IQR, 6287‐7200) on D7 (*P* = .004) and peak thrombin (D0 = 5.7 × 10^10^ ± 0.1 vs D7 = 4.4 × 10^10^ ± 1.5; *P* = .03; Figure 5A,B). However, only DAT significantly prolonged *T*_max_ on D7 compared to clopidogrel treatment on D7 (1980 s; IQR, 1380‐4067; *P* = .004). Dual antithrombotic treatment also decreased thrombin generation potential or AUC (D0 = 6.2 × 10^10^ ± 1.4 vs D7 = 3.3 × 10^10^ ± 1.8; *P* = .01) as well as *V*_max_ (D0 = 23 000 RFU/s; IQR, 20 750‐32 000 vs D7 = 8570 RFU/s; IQR, 4340‐10 756; *P* = .004; Figure 5C,D). Both DAT and rivaroxaban treatment on D7 caused a significant decrease in *V*_max_ compared to clopidogrel on D7 (30 000 RFU/s; IQR, 18 000‐41 500; *P* = .0004 and *P* = .005, respectively). As expected, clopidogrel treatment did not significantly alter any of the thrombin generation variables as shown in Figure 5B.

### 3.4 Correlations between plasma anti‐Xa levels and platelet‐dependent thrombin generation

A strong and significant negative correlation was found between plasma rivaroxaban concentrations and change (%) in *V*_max_ in thrombin generation before and after rivaroxaban treatment. After 7 days of DAT, both peak thrombin generation and *V*_max_ were strongly and negatively correlated with plasma rivaroxaban concentrations. Data are summarized in Table 2.

**TABLE 2:** Correlation between plasma rivaroxaban concentrations and changes in platelet dependent thrombin generation.

	Change (%) in thrombin generation			
	AUC	*T*_max_	Peak Thrombin	*V*_max_
	−45.8 (IQR: −74.2 to 28.5)	346 (IQR: 41.4 to 1006)	−10.8 (IQR: −40.8 to −3.3)	−51.7 (IQR: −78.8 to 25.6)
RIVA Anti‐Xa	*R* = 0.39	*R* = 0.18	*R* = −0.65	*R* = −0.78
*P*‐value	.39	.64	.067	.0017
	−61.3 (IQR: −70.0 to −10.9)	133 (IQR: 111.4 to 239.4)	−78.2 (IQR: −87.6 to −70.0)	−75.3 (IQR: −85.9 to 53.7)
DAT Anti‐Xa	*R* = −0.23	*R* = 0.14	*R* = −0.84	*R* = −0.84
*P*‐value	.56	.14	.0070	.0070

Abbreviations: AUC, area under the curve; RIVA, rivaroxaban; *T*_max_, time to maximum thrombin generation; *V*_max_, maximum velocity.

## 4 DISCUSSION

We found a synergistic inhibitory effect of clopidogrel and rivaroxaban on platelet activation and thrombin generation potential in feline platelets. Specifically, DAT with clopidogrel and rivaroxaban safely decreased platelet reactivity to thrombin activation more effectively than clopidogrel alone. A dosage of 18.75 mg clopidogrel and 2.5 mg rivaroxaban PO given q24h for 7 days was well‐tolerated in healthy cats without any adverse effects.

Platelet activation, in the presence or absence of physiological agonists, was measured by upregulation of platelet surface P‐selectin, a marker of outside‐in signaling that leads to alpha granule secretion. In addition, tissue factor‐induced thrombin generation was measured in PRP to evaluate the pharmacodynamic effects of DAT on the link between primary and secondary hemostasis. Our data indicate that DAT in cats not only decreased the number of activated platelets but also modulated their thrombin generation potential. A plausible mechanism of these findings is that rivaroxaban, a direct factor Xa inhibitor, modulates the activation of thrombin during the initial phase of coagulation when tissue factor and activated factor VII complex activate factors X and V. Evidence in human platelets suggests that upon activation, platelets undergo de novo synthesis of tissue factor leading to its subsequent externalization and interaction with extracellular factor VIIa.^27^ Additional antagonism of the ADP receptor, P2Y_12_, by clopidogrel may further dampen thrombin generation by downregulating platelet‐derived tissue factor synthesis and release. This combined dampening of thrombin generation during the initial phase of coagulation may explain the decreased number of activated platelets and rate of thrombin generation. The synergistic inhibitory effect of clopidogrel and rivaroxaban on the number of activated platelets and platelet‐dependent thrombin generation may have clinical relevance in cats. Although increased platelet activation previously has been observed in cats with HCM and other cardiomyopathies, its clinical and prognostic importance is unclear. In human beings, a persistent increase in activated platelets is associated with acute myocardial infarction and increased risk of recurrent thrombosis.^28^ Hence, thromboprophylaxis with DAT may be superior to mono‐agent treatment in preventing arterial thromboembolism and recurrent thrombosis in clinical HCM by lowering the numbers of activated platelets. Future randomized clinical trials are needed to test this hypothesis.

Upon treatment of PRP with supraphysiologic amounts of tissue factor, DAT was more effective at suppressing thrombin generation than rivaroxaban treatment alone. These findings are in contrast to studies in humans that showed that rivaroxaban administration modulates thrombin generation potential in tissue factor‐treated plasma.^29^ This may be a result of assay and species differences. Central to the procoagulant properties of platelets is the expression of phosphatidylserine on the external leaflet of the platelet membrane. During the propagation phase of coagulation, prothrombinase complex (Fxa and Fva), which forms on exposed phosphatidylserine, catalyzes a thrombin burst leading to the subsequent conversion of fibrinogen to fibrin and clot formation.^29^ The externalization of phosphatidylserine is facilitated by the enzyme scramblase upon platelet stimulation.^30^ This enhanced antithrombotic potency by DAT that was not seen in single agent treatment with rivaroxaban suggests that multi‐pathway inhibition may play a role in suppressing scramblase activation. Irreversible inhibition of P2Y_12_ by clopidogrel and indirect inhibition of thrombin‐mediated activation by rivaroxaban likely modulate cytoplasmic calcium mobilization to suppress scramblase activation.^31^ This hypothesis was further supported by strong negative correlations between plasma rivaroxaban concentrations and changes in peak thrombin generation and thrombin generation kinetics after DAT despite achieving subtherapeutic concentrations of rivaroxaban with our dosing regimen. This finding also suggests that a higher dose may be required to achieve more marked suppression of thrombin generation when rivaroxaban is given alone. Additional studies are needed to delineate the mechanisms of this synergistic inhibitory effect of DAT on platelet‐dependent thrombin generation.

Thrombin propagation by activated platelets is partially dependent on their reactivity to physiologic agonists.^32^ Consistent with our thrombin generation data, platelet reactivity to ADP and thrombin was significantly decreased after DAT. As expected and based on its mechanism of action, clopidogrel decreased reactivity to ADP but did not elicit a consistent modulatory effect on thrombin‐mediated platelet activation in all cats. This finding is important because thrombin is the most potent physiologic agonist that elicits strong outside‐in signaling, leading to marked platelet activation, calcium mobilization, integrin activation and platelet aggregation via the protease activating receptor (PAR) and co‐receptor of GP1bα.^32^ Thrombin‐induced platelet reactivity plays a crucial role in arterial thrombosis in animal models and has been demonstrated in young adults with ischemic stroke.^33^ Large‐scale clinical studies are needed to confirm the clinical relevance and benefits of DAT on thrombin‐mediated platelet activation in cats. The underlying mechanisms of DAT‐mediated attenuation in platelet reactivity to thrombin, however, remain unclear. A previous study found that in vitro treatment of human platelets with rivaroxaban and aspirin resulted in similar modulation in reactivity to the PAR‐1 activating peptide, TRAP‐6, but no effects were observed in the presence of thrombin.^34^ This discrepancy may be because of species differences because PAR receptor subtypes (1‐4) have not been characterized in feline platelets. In addition, it is plausible that the PAR‐1 receptor may play a more prominent role in mediating platelet activation in cats because FXa has been shown to directly activate platelets via PAR‐1 in human platelets.^35^

Although both DAT and clopidogrel treatment attenuated platelet aggregation in response to ADP, DAT did not further diminish platelet aggregation compared to clopidogrel treatment alone. Our findings correspond with previous studies that have shown that a standard dose of clopidogrel results in significant inhibition of ADP‐induced platelet aggregation.^11^ Additional studies are needed to determine if cats with the genetic polymorphism in *P2RY1*, which is associated with clopidogrel resistance, would benefit from DAT. Interestingly, rivaroxaban potentiated platelet activation and aggregation in response to ADP. This finding differs from studies in humans and other animal models showing that rivaroxaban alone has minimal impact on ADP‐induced platelet aggregation.^36^ A plausible explanation is that in vitro platelet activation, which commonly occurs in feline platelets, could dampen their reactivity to ADP before treatment.^37^ Rivaroxaban treatment may therefore lower pre‐activation of platelets at rest, resulting in potentiated activation and aggregation in the presence of physiologic agonists in vitro. However, because no significant differences in P‐selectin positive platelets were found before and after rivaroxaban treatment, an underlying platelet priming mechanism mediated by rivaroxaban causing unrestrained platelet aggregation and activation also is possible. The mechanism by which rivaroxaban primes feline platelets to ADP is unclear and further research is warranted.

Our study had several limitations. First, the small sample size and 7‐day duration of each treatment may limit the clinical recognition of adverse events that are less frequent or take longer to be manifested. Second, ADP was the only agonist used for the platelet aggregation assay. Utilization of different agonists such as arachidonic acid, tissue factor or collagen may have identified differences among treatments on platelet aggregation. Third, predetermined doses were given without dose adjustments for weight. Although this design accurately represents clinical usage of these drugs, the variable dosages may have resulted in large SDs in drug effect on platelet function. However, this concern may not be an important limitation because many cats were of similar body weight. Finally, the cats included in ours study were all healthy and selected from a research colony of related cats. This feature may affect our ability to extrapolate the physiological response to DAT to the general population of cats, and in hypercoagulable cats with HCM. Future prospective clinical trials examining dual clopidogrel and rivaroxaban treatment in a large heterogeneous population of cats with CATE or HCM are needed.

Overall, we found that DAT with clopidogrel and rivaroxaban lowered the number of activated circulating platelets, dampened platelet response to thrombin, and suppressed tissue‐factor‐induced thrombin generation in healthy cats more effectively than monotherapy with clopidogrel or rivaroxaban alone. The potentiated response to ADP in platelets secondary to rivaroxaban requires further investigation. Because cats with severe HCM have increased platelet activation and increased platelet priming, randomized clinical trials are needed to determine if DAT is superior to monotherapy in preventing intracardiac thrombosis and CATE in cats with HCM.^11^ Additionally, we did not characterize the ADP receptor genes *P2RY1* and *P2RY12* in our small population of cats to determine which cats could have been genetically resistant to clopidogrel's platelet inhibition effects.^14^ Our study suggests that DAT with clopidogrel and rivaroxaban holds promise for thromboprophylaxis in cats with cardiomyopathy and that clinical trials in such patients are warranted.

## CONFLICT OF INTEREST DECLARATION

Authors declare no conflict of interest.

## OFF‐LABEL ANTIMICROBIAL DECLARATION

Authors declare no off‐label use of antimicrobials.

## INSTITUTIONAL ANIMAL CARE AND USE COMMITTEE (IACUC) OR OTHER APPROVAL DECLARATION

Approved by the IACUC of University of California, Davis, number 20565.

## HUMAN ETHICS APPROVAL DECLARATION

Authors declare human ethics approval was not needed for this study.
